# Detection of hydrophobicity grade of insulators based on AHC-YOLO algorithm

**DOI:** 10.1038/s41598-025-92696-0

**Published:** 2025-03-20

**Authors:** Shaotong Pei, Weiqi Wang, Peng Wu, Chenlong Hu, Haichao Sun, Keyu Li, Mianxiao Wu, Bo Lan

**Affiliations:** Hebei Provincial Key Laboratory of Power Transmission Equipment Security Defense, North China Electric Power University, 619 Yonghuabei Street, Baoding City, People’s Republic of China

**Keywords:** Composite insulators, Hydrophobicity, Defect detection classification, YOLO, Engineering, Electrical and electronic engineering

## Abstract

Thanks to the rapid development of image processing technology, the efficiency and accuracy of power inspection have been enhanced through deep learning techniques. However, during on-site inspections, the complexity of the background images of composite insulators often makes it difficult to directly extract key features for accurately assessing hydrophobicity levels. Moreover, considering the real-time requirements for insulator hydrophobicity detection in practical operations, the model must be lightweight to speed up the detection process. To address this issue, this paper proposes a YOLO algorithm for the precise detection of composite insulator hydrophobicity. The algorithm integrates a high-performance GPU network (HGNetv2), a mixed local channel attention mechanism (MLCA), lightweight convolution (CSPPC), and the Inner-WIoU loss function, significantly reducing the network’s burden and improving the accuracy of recognizing composite insulator sheds and classifying their hydrophobicity levels. By adopting a strategy of identifying insulator sheds and then classifying their hydrophobicity levels, precise detection of hydrophobicity is achieved. Experimental results show that the proposed AHC-YOLO algorithm has increased the detection accuracy of sheds by 5.77%, with GFLOPs reduced to 5.8. In the task of classifying hydrophobicity levels, the Top-1 accuracy has been improved by 4.994%, with GFLOPs reduced to 1.9. These achievements not only meet the needs for the detection and classification of composite insulator hydrophobicity but also further demonstrate the effectiveness and superiority of the algorithm through ablation and comparative experiments.

## Introduction

Composite insulators, which consist of a core made of glass fiber-reinforced epoxy resin and an outer insulating layer typically made of silicone rubber, are increasingly being used in power transmission systems due to their excellent anti-pollution performance and lightweight nature. However, over time, factors such as corona discharge and environmental influences can cause aging of the insulator sheds, posing potential threats to the safety of the power grid. To ensure the secure and reliable operation of transmission lines, it is necessary to conduct regular hydrophobicity tests on composite insulators and promptly decommission those that have severely aged based on the test results^1–4^.

The hydrophobicity of insulators refers to their ability to resist the penetration of water and moisture on their surface. This characteristic is crucial for the performance of insulators in power systems as it directly affects their insulating properties in damp conditions. Hydrophobicity prevents the formation of continuous water films on the insulator surface, thereby limiting surface leakage currents and enhancing the flashover voltage. Therefore, monitoring hydrophobicity allows for timely assessment of an insulator’s ability to resist contamination flashovers^5–7^.

Common methods for assessing the hydrophobic performance of composite insulators include the contact angle method, surface tension method, and water spray grading method. The contact angle and surface tension methods are simple and quantitatively accurate for measurements but require a controlled experimental environment, making them suitable for laboratory studies. The water spray grading method involves spraying water mist and observing the distribution of water droplets on the sample surface, comparing it with grading criteria and standard images to determine the sample’s hydrophobicity. However, it still necessitates on-site pole climbing by maintenance personnel^8–10^.

Thanks to the rapid advancement of drone and artificial intelligence technologies, the application of AI algorithms has made it possible to automatically recognize and classify defects from vast amounts of image data, enhancing the efficiency and accuracy of inspections. Through deep learning techniques, systems can continuously learn and optimize to adapt to various environments and conditions, further improving recognition accuracy.At present, an automated inspection model has been established, primarily based on machine inspection, supplemented by manual inspections^11–13^.

Experts have integrated hydrophobicity detection and assessment with advanced image processing and drone technologies, achieving certain successes in replacing manual inspections. For instance, Zhou Lijun and colleagues^14^developed a technology that integrates an improved Canny edge detection algorithm with deep residual networks for the identification of insulator hydrophobicity. This technology first employs the enhanced Canny algorithm to capture the edges of water droplets on insulator sheds, reducing the interference of color and lighting variations on the recognition results. Subsequently, deep residual networks (ResNet101) are utilized for transfer learning, and deformable convolutional networks (DCN) are incorporated to enhance the model’s adaptability to variations in water droplet shapes. Experimental results indicate that this method achieves a recognition accuracy of up to 92.9%. Yang Qiuyu and others^8^ proposed an intelligent method for identifying the hydrophobicity of composite insulators. This method uses image processing techniques to first perform histogram equalization and filtering on the images, followed by Otsu threshold segmentation to separate water droplets/traces. By calculating features such as the coverage ratio, area ratio, and shape factor of water droplets/traces, and applying an SVM to build a classification model, the method achieves automatic recognition of composite insulator hydrophobicity. Tests show that this approach can accurately identify seven different hydrophobicity levels with an average accuracy exceeding 80%. However, there are still some problems with the above studies and methods, such as the complex background of composite insulator images in practical applications, which is affected by a variety of factors. It leads to difficulties in directly extracting the key features for accurately assessing the hydrophobicity grade, which limits the accuracy of detection. And in order to apply to the edge end, it is necessary to maintain a balance between accuracy and light weight.

In response to the challenges identified in previous studies and the need for precise detection and classification of composite insulator hydrophobicity, this paper has improved the YOLOv8^15^algorithm (You Only Look Once). We propose an Accurate testing of hydrophobicity of composite insulators YOLO (AHC-YOLO) algorithm. The AHC-YOLO algorithm is based on the YOLOv8 algorithm and incorporates a high-performance GPU network (High Performance GPU Network V2, HGNetv2)^16^and a mixed local channel attention mechanism (MLCA)^17^into the backbone network to significantly reduce its computational load while maintaining computational volume and enhancing the network’s detection and classification accuracy. A lightweight convolution (CSP-PartialConv, CSPPC)^18^is introduced in the Head part to reduce the processing of redundant information and maintain the network’s lightweight nature. Finally, the Inner-WIoU loss function^19^ is adopted to further improve the accuracy of detecting composite insulator sheds and classifying their hydrophobicity levels.

This paper divides the detection and classification of composite insulator hydrophobicity into two parts. First, the AHC-YOLO algorithm is used for target detection of insulator sheds, and then the water trace area on the sheds can be extracted based on the position of the bounding box and the cropping coefficient. Finally, the extracted water trace area images are classified again through the AHC-YOLO algorithm to determine the hydrophobicity level of the insulator. The model, when equipped with a drone, can perform real-time detection of the hydrophobicity of composite insulators in the field, showing a promising application prospect. The dataset is constructed through two approaches: spraying and shooting of retired and aged composite insulators on-site with a drone, and crawling network resources. For the above research work, this paper introduces the composition and principle of the AHC-YOLO algorithm proposed in this paper through the AHC-YOLO Algorithm Principle section; the acquisition and composition of the relevant dataset through the Test Dataset Generation section; the effectiveness of the algorithm is evaluated through the Experimental Results and Analysis section; and, finally, the results of this research are summarized through the Conclusion section.

## AHC-YOLO algorithm principle

The AHC-YOLO algorithm is based on YOLOv8 and is improved by introducing the HGNetv2 network, the MLCA attention mechanism, the CSPPC lightweight convolutional structure, and the Inner-WIoU loss function.

### YOLOv8 algorithm principle

YOLOv8 is a state-of-the-art object detection algorithm that offers enhanced accuracy and detection speed compared to its predecessors^20–27^. The network structure of YOLOv8 is primarily composed of three parts: the Input section, which feeds composite insulator images into the network; the Backbone module, responsible for recognizing and extracting features from the composite insulator images; and the Head section, which pools and fuses the features extracted by the Backbone and outputs the results.

In YOLOv8, the network structure has undergone several optimizations compared to previous models. These improvements include a more efficient architectural design, a more refined training process, and enhanced feature extraction capabilities. Particularly in the Backbone section, the original C3 module from YOLOv5 has been replaced with a new C2f structure. Additionally, the number of channels in models of different sizes has been adjusted to reduce computational complexity and further lightweight the model. In the Head section, YOLOv8 employs a decoupled head structure, replacing the coupled head structure from YOLOv5, separating classification and detection tasks, and transitioning from Anchor-Based detection to Anchor-Free, which not only improves the ability to detect objects but also shows advantages in recognizing irregularly shaped targets. Furthermore, YOLOv8 introduces a positive and negative sample matching strategy in the computation of the loss function, combining Distribution Focal Loss and Complete Intersection over Union (CIoU) Loss as regression losses, which helps to more effectively address background and class imbalance issues, enabling the network to more rapidly identify the distribution around the target location.

### AHC-YOLO algorithm

**Fig. 1:**
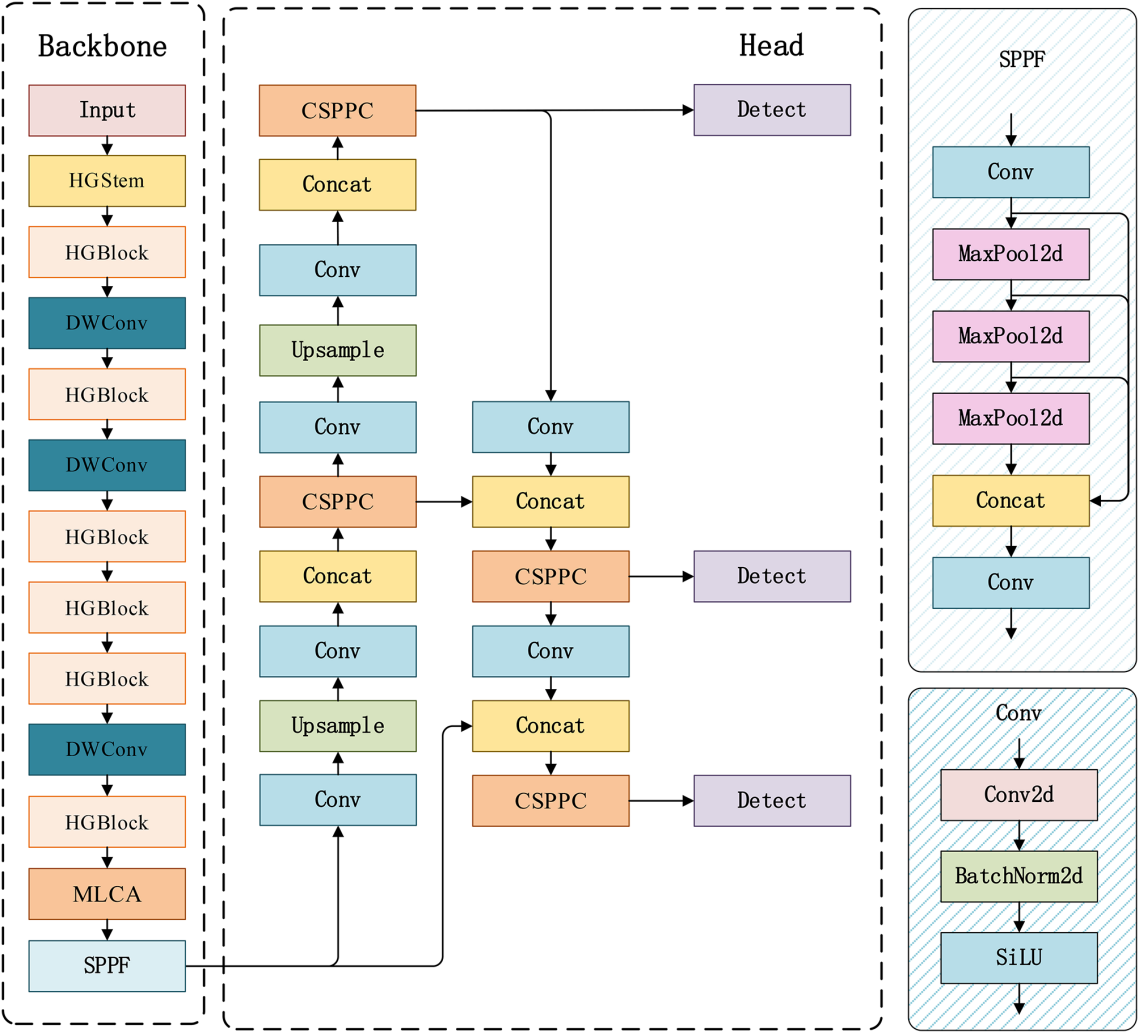
AHC -YOLO structure.

The AHC-YOLO algorithm, based on YOLOv8, has been developed to meet the precise detection requirements for the hydrophobicity of composite insulators as outlined in this paper, with four key enhancements incorporated. Firstly, to ensure the scalability of the algorithm for edge applications, the HGNetv2 network is integrated, significantly reducing the algorithm’s complexity and computational load while maintaining network performance. Subsequently, to enhance the precision of the AHC-YOLO algorithm in identifying the sheds of composite insulators, the MLCA (Mixed Local Channel Attention) mechanism is employed. The MLCA mechanism, which combines local average pooling with global average pooling, reduces the parameter count and computational complexity, thereby substantially improving detection accuracy with a minimal increase in parameter volume. Additionally, to effectively reduce unnecessary computations in feature fusion and address redundant information in feature maps, the CSPPC (CSP-PartialConv) lightweight convolutional structure is utilized to refine the C2f module. This modification decreases the network’s computational complexity while preserving the model’s performance. Finally, the Inner-WIoU loss function is implemented to further enhance the accuracy of detecting composite insulator sheds. By improving the accuracy of the bounding box for shed detection, it indirectly affects the accuracy of hydrophobicity level classification. The structure of the AHC-YOLO algorithm is depicted in Fig. 1.

### HGNetV2 network

The HGNetv2 network is a high-performance backbone network designed by Baidu’s PaddlePaddle Vision Team, which excels in multi-label classification and object detection tasks. To address the need for strong feature recognition capabilities in hydrophobicity level detection and classification, which often leads to complex structures and high computational loads, this paper selects the HGNetV2 network structure as the feature extraction network while ensuring algorithm precision and lightweight design.

**Fig. 2:**
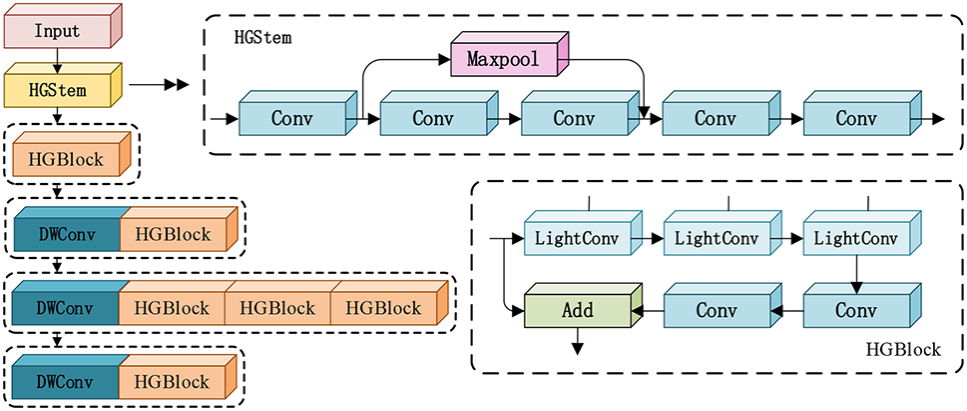
HGNetv2 network and its constituent modules.

HGNetV2 (High Performance GPU Network V2), consists of three main components: the HStem, HGBlock, and Depthwise Separable Convolution (DWConv). The HGNetv2 network structure is depicted in Fig. 2. This network is known for its efficient architectural design, which increases the number of standard convolutions with the depth of layers, resulting in a backbone network that is favorable for GPU inference. It not only surpasses other CNNs in accuracy at the same speed but also offers better cost-performance compared to the ViT-base model.

The HGStem layer serves as the initial preprocessing stage of the network, with its primary task being the extraction of key features from the input data. As shown in Fig. 2, the HGStem structure begins with a 3 × 3 convolutional operation. The data is then split into two branches: one path performs a max pooling operation, while the other path undergoes a 2 × 2 convolution. Both operations aim to reduce the number of channels while maintaining the spatial dimensions of the data. After dimension padding, a 2 × 2 convolution is applied again to restore the number of channels and halve the size of the feature maps. The two branches are then merged, and two consecutive convolutional operations are performed to reduce the size and number of channels of the feature maps, effectively alleviating the computational burden on the subsequent network.

The HGBlock module is composed of standard convolutions and lightweight convolutions (Lightweight Convolution, LightConv). The structure of the HGBlock is depicted in Fig. 2. A Depthwise Separable Convolution (DWConv) is employed at the forefront of the HGBlock. This enhances the model’s inference speed, effectively reduces the model’s parameter count, and helps mitigate the risk of overfitting, thereby improving the model’s generalization capabilities. It is particularly suitable for deployment in resource-constrained environments, such as edge computing scenarios.

### MLCA attention mechanism

The MLCA (Mixed Local Channel Attention) mechanism is designed to enhance the efficiency and performance of algorithms, particularly when dealing with complex backgrounds in composite insulator images captured on-site, which can adversely affect the recognition of insulator shed features. To effectively improve algorithmic efficiency and performance, the MLCA attention mechanism is employed to shift the network’s focus towards the insulator sheds. This mechanism not only maintains good performance but also minimizes the additional computational load introduced by the algorithm. The MLCA is a lightweight attention mechanism that combines local average pooling and global average pooling, reducing the number of parameters and computational complexity while significantly enhancing detection accuracy with a minimal increase in parameter volume.

**Fig. 3:**
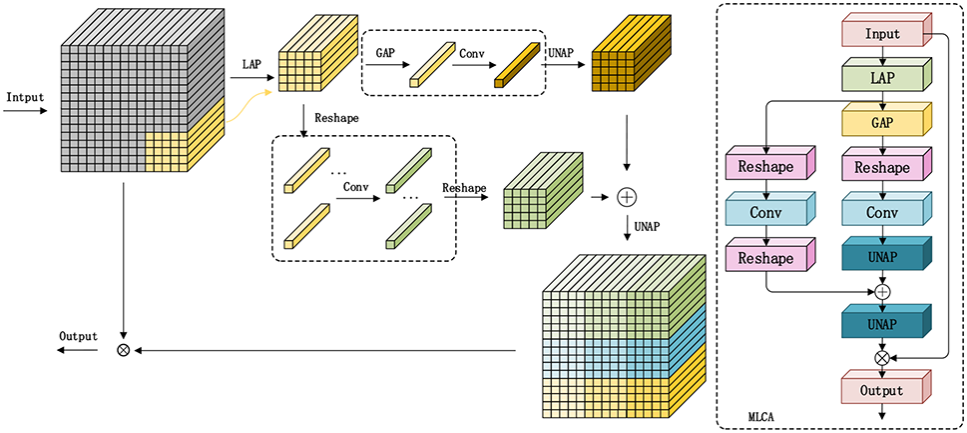
Structure and working principle of MLAC attention mechanism.

Figure 3 shows the structure and working principle of Mixed Local Channel Attention (MLCA). Where LAP is Local Average Pooling, GAP is Global Average Pooling, UNAP is Inverse Pooling Operation and Reshape is Rearranging Features. The MLCA attention mechanism initially applies LAP to the feature maps to capture local features. It then conducts convolutional operations on these features, as well as on features that have undergone global average pooling. The features that have been globally pooled are convolved, reshaped, and unpooled before being combined with the locally pooled features, achieving an integration of global feature information. After unpooling, these features are merged with the original input features, thereby enhancing the model’s focus on key features.

To improve the feature extraction efficiency of the AHC-YOLO algorithm and to strengthen the model’s feature extraction capabilities, this paper selects the MLCA attention mechanism to be incorporated into the feature extraction part of YOLOv8. This enhancement aims to improve the detection of composite insulator sheds and the classification accuracy of hydrophobicity levels.

### CSPPC lightweight convolutional structure

In the context of drone edge applications, the operational speed and lightweight design of the AHC-YOLO algorithm are of paramount importance. Therefore, the original structure of YOLOv8 has been optimized to achieve more efficient computation. This paper employs a lightweight convolutional structure that combines the CSP structure with PartialConv, replacing the C2f module in YOLOv8 and effectively reducing computational complexity.

**Fig. 4:**
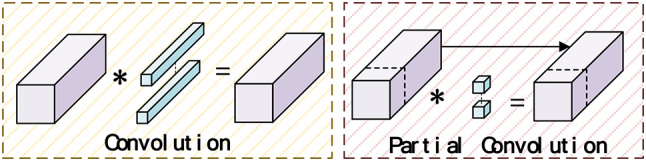
Conventional Convolution and PConv Structures.

PartialConvolution (PConv) structure is a lightweight convolutional neural network design that computes features for only a subset of channels and passes the remaining channels through identity mappings. By leveraging redundant information in feature maps, it eliminates unnecessary computational steps. Often, in the multiple channels of a network, there is a significant amount of similar or repeated data that is processed repeatedly without adding new useful features, leading to a waste of computational resources and memory access. The PartialConv structure optimizes this process, enhancing the network’s efficiency and performance. Its specific structure is shown in Fig. 4.

As depicted in Fig. 4, the primary distinction between PConv and traditional convolutional methods is that PConv applies conventional spatial feature extraction convolution operations only to a subset of the input channels, leaving the remaining channels unaltered. This strategy effectively simplifies the overall computational process.

**Fig. 5:**
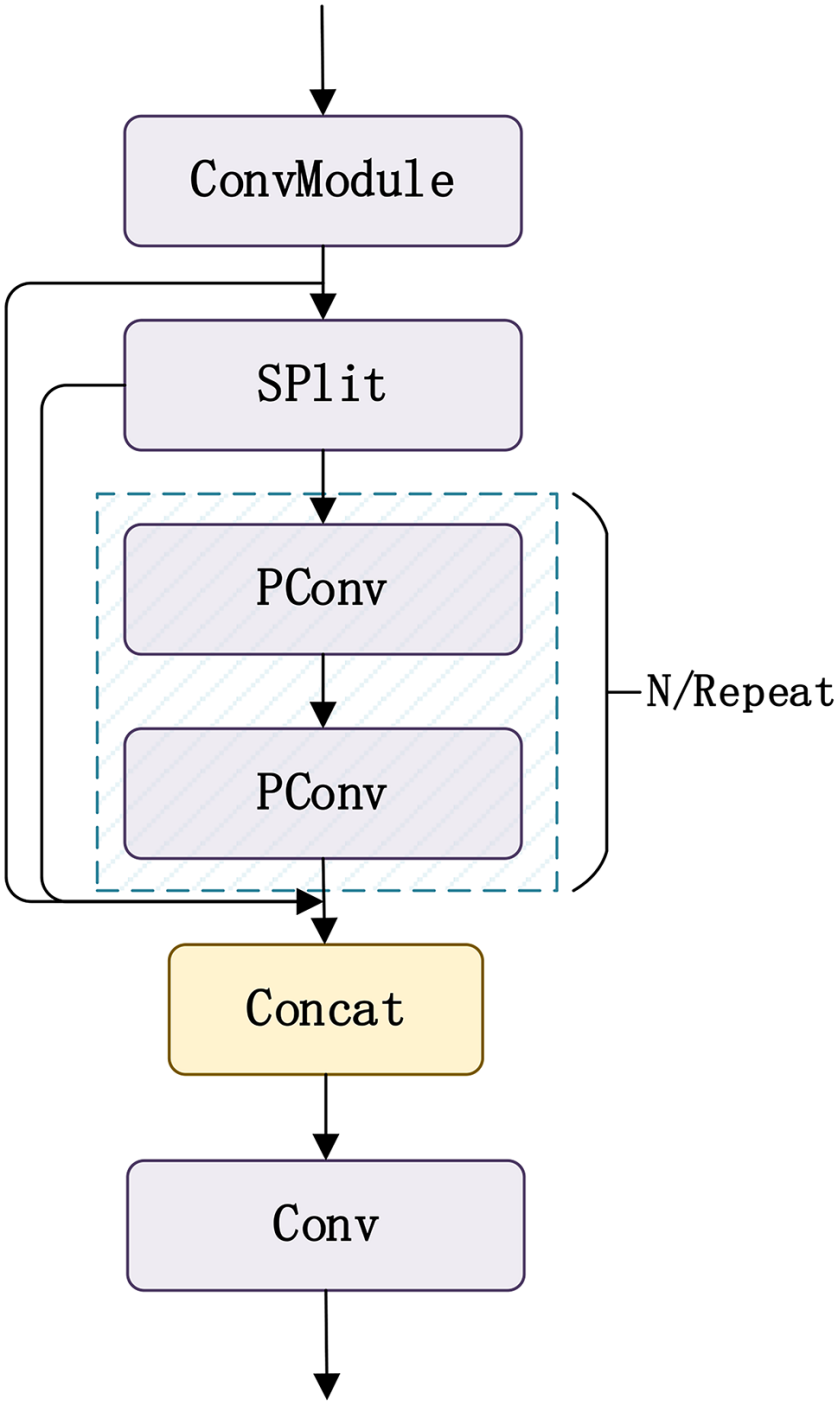
CSPPC structure.

As shown in Fig. 5, the CSPPC structure is a combination of the CSP structure and the PartialConv structure. This paper employs the CSPPC structure to replace the C2f module, ensuring algorithm performance while making the model more lightweight.

### Inner-WIoU loss function

Given the necessity for hydrophobicity classification to rely on the precision of composite insulator shed detection, the loss function not only affects the accuracy of shed detection but also indirectly impacts the precision of hydrophobicity level classification through its influence on the accuracy of the bounding box. Therefore, the AHC-YOLO algorithm proposed in this paper employs the Inner-WIoU loss function to improve upon the CIoU loss function used in the original YOLOv8. Existing IoU-based boundary regression methods primarily accelerate convergence by adding new loss terms, yet overlook the inherent limitations of the IoU loss term itself. These methods lack the self-adjustment capability to adapt to different detectors and detection tasks in practical applications, leading to poor generalization. To address this, this paper introduces the Inner-IoU Loss, which calculates the IoU loss with the aid of auxiliary boundaries and incorporates a scaling factor, ratio, to adjust the size of the auxiliary boundaries, thereby more accurately computing the loss. Building upon the Inner-IoU, this paper further integrates a similarity metric to form the Inner-WIoU loss function. This improvement not only effectively reduces fluctuations during training but also optimizes the model’s detection capability for targets of varying sizes by assigning higher weights to smaller targets, achieving a balance in detection performance.

**Fig. 6:**
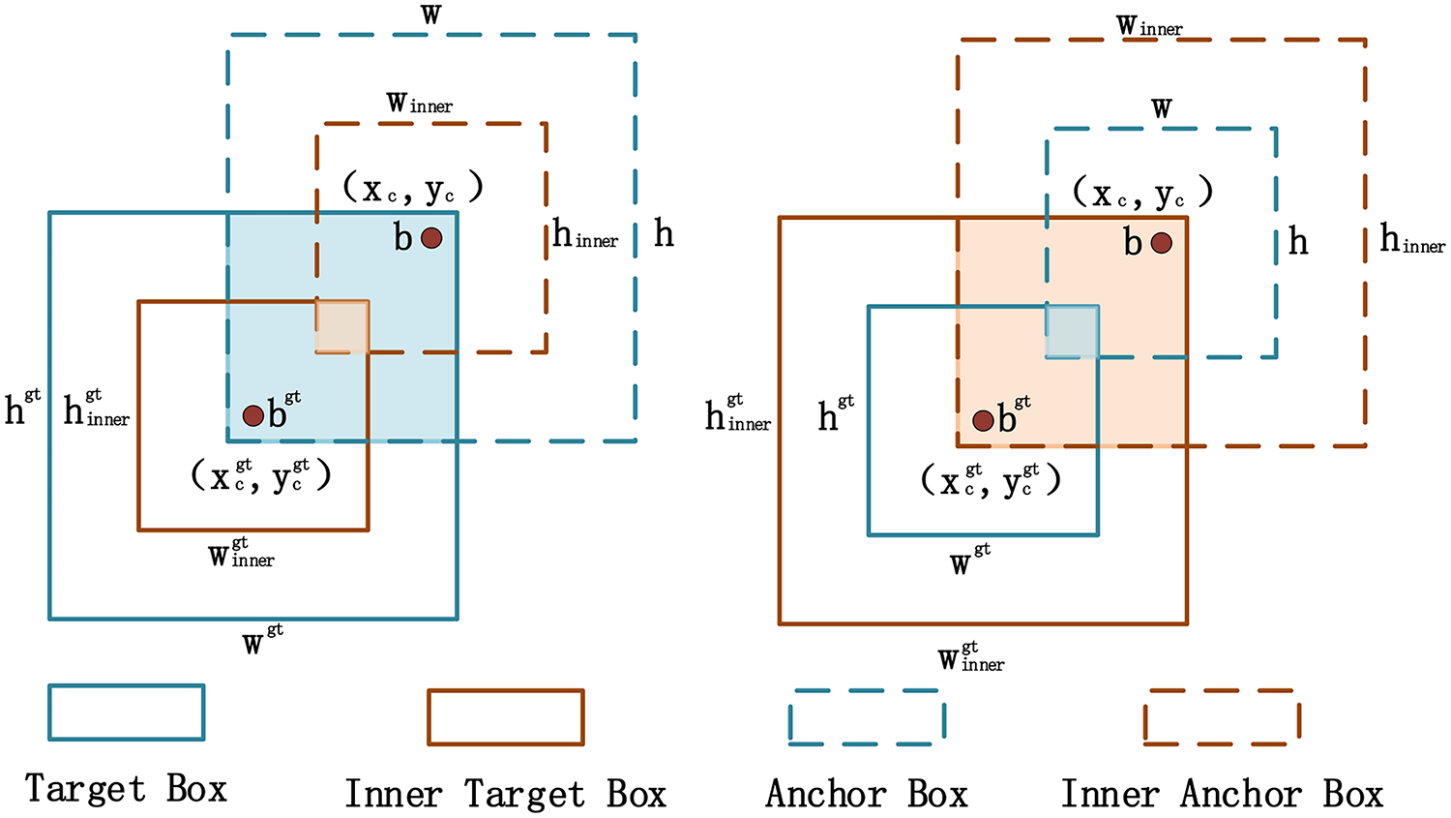
Inner-IoU depiction.

As shown in Fig. 6, B^gt^ and B represent the ground truth bounding box (GT box) and the anchor box, respectively. The center coordinates of the ground truth bounding box are (x_c_^gt^, y_c_^gt^), while those of the anchor box are (x_c_, y_c_). The width and height of the ground truth bounding box are denoted by w^gt^ and h^gt^, whereas the width and height of the anchor box are represented by w and h. The scale factor “ratio” typically ranges from [0.5, 1.5]. The core concept of Inner-IoU is to enhance the precision of bounding box regression (BBR) in object detection, particularly effective when dealing with highly overlapping objects. Unlike the conventional IoU (Intersection over Union) calculation method, which considers the overall overlap between the predicted bounding box and the ground truth bounding box, Inner-IoU focuses more on the overlap within the bounding box itself. It calculates the loss function by introducing an auxiliary boundary, thereby optimizing the accuracy of BBR.

WIoU, through a dynamic non-monotonic focusing mechanism, is capable of focusing on general quality anchor boxes, thereby enhancing the overall performance of the detector. WIoU can also dynamically adjust the focus on low-quality examples, making it more generalized across datasets of varying qualities. Moreover, the WIoU loss function introduces a weight factor that allows for different levels of weighting for objects of different classes, addressing the issue of class imbalance. It is particularly advantageous for multi-class detection. Therefore, the Inner-IoU is applied to the WIoU-based bounding box regression loss function, resulting in the Inner-WIoU, which is defined as follows:

1 $$b_{l}^{{gt}}=x_{c}^{{gt}} - \frac{{{w^{gt}} \cdot ratio}}{2},b_{r}^{{gt}}=x_{c}^{{gt}}+\frac{{{w^{gt}} \cdot ratio}}{2}$$

2 $$b_{t}^{{gt}}=y_{c}^{{gt}} - \frac{{{h^{gt}} \cdot ratio}}{2},b_{b}^{{gt}}=y_{c}^{{gt}}+\frac{{{h^{gt}} \cdot ratio}}{2}$$

3 $${b_l}={x_c} - \frac{{w \cdot ratio}}{2},{b_r}={x_c}+\frac{{w \cdot ratio}}{2}$$

4 $${b_t}={y_c} - \frac{{h \cdot ratio}}{2},{b_b}={y_c}+\frac{{h \cdot ratio}}{2}$$

5 $$inter=(\hbox{min}(b_{\tau }^{{gt}},{b_\tau })\,-\,\hbox{max}(b_{l}^{{gt}},{b_l}))\,\cdot\,(\hbox{min}(b_{b}^{{gt}},{b_b})\,-\,\hbox{max}(b_{t}^{{gt}},{b_t}))$$

6 $$union=({w^{gt}}\,\cdot\,{h^{gt}})\,\cdot\,{(ratio)^2}\,+\,(w\,\cdot\,h)\,\cdot\,{(ratio)^2}\,-\,inter$$

7 $$Io{u^{inner}}=\frac{{inter}}{{union}}$$

8 $${L_{Inner - WIoU}}={L_{WIoU}}+IoU - Io{U^{inner}}$$

## Testing dataset generation

To train the algorithm to recognize the hydrophobicity of composite insulators in real environments, the dataset was chosen to be captured in the field as well as obtained from web resources.

### Classification standards for composite insulator hydrophobicity

According to the national standard GB/T 24,622 − 2022 “Guidelines for the Measurement of Hydrophobicity on Insulator Surfaces,” the hydrophobicity of composite insulators is categorized into seven grades, ranging from HC_1_ to HC_7_. These grades indicate a progressive decrease in hydrophobic performance, from the high hydrophobicity of HC_1_ to the low hydrophobicity of HC_7_ .

### Composite insulator shed dataset

To meet the requirements for the detection of composite insulator sheds, this paper has constructed a dataset of composite insulator sheds. The dataset comprises 7960 valid sample photos, some of which were obtained through on-site shooting, while others were collected from online resources. During the field shooting process, we utilized drones to spray water and take photos of disconnected composite insulator strings to acquire image data. Due to the larger visible area of large sheds compared to small ones, and the fact that water traces are easier to detect and observe, we focused our shooting on the large sheds of composite insulators. Considering that the upper surface of large sheds usually shows more severe aging than the lower surface, and the test results are more consistent, we concentrated on the large sheds of composite insulators during shooting. Then, we used the LabelImg tool to accurately label the positions of the large shed surfaces in these photos. To train and validate the model, we divided the dataset into training, validation, and testing sets in an 8:1:1 ratio.

To ensure the accuracy of the labeling, we followed the group standard T/SDPEA 0015–2019 “Labeling Specification for Image Data Set of Hidden Danger/Defect Target Recognition in Power Transmission and Transformation Inspection” released by the Shandong Electric Power Enterprise Association in 2019. We required the labeling personnel to conduct self-inspection first, followed by a review by professional reviewers to ensure the high quality of the dataset labeling.

## Experimental results and analysis

In order to verify the effectiveness of the AHC-YOLO algorithm, the experimental part was chosen to demonstrate the superiority of the present algorithm through ablation, comparison experiments, and heat maps.

### Experimental setup

During the training of the AHC-YOLO algorithm for shed target detection and hydrophobicity classification, the following configurations were used:

The GPU utilized was the NVIDIA GeForce GTX 3090, while the CPU was the Intel(R) Xeon(R) CPU E5-2680 v4 with a base frequency of 2.40 GHz. In terms of software environment, Python 3.9.0 was used, paired with the PyTorch framework version 1.10.0, and CUDA version 11.3.1.

Regarding the hyperparameter settings during training: firstly, the batch size was set to adjust automatically to maintain the GPU memory usage around 60%; secondly, the maximum number of training iterations was set to 3000 rounds, and then the patience parameter was set to 200. This means that if there is no significant improvement in the validation metrics for 200 consecutive training rounds, it is considered that the model performance has stabilized, and training will be halted to prevent model overfitting. Finally, the SGD (Stochastic Gradient Descent) optimizer was chosen for the model.

To demonstrate the effectiveness of the insulator low-value defect recognition algorithm, this paper measures the detection performance of the model using two metrics: Mean Average Precision (MAP) and Giga Floating-point Operations Per Second (GFLOPs). MAP is a commonly used indicator to evaluate the overall performance of an algorithm model, and its calculation formula is defined as follows:

9 $${\text{mAP}}=\frac{1}{N}\sum\limits_{{i=1}}^{N} {{t_{{\text{AP}}i}}}$$

10 $${\text{AP}}=\sum\limits_{{i=1}}^{n} {\Delta R_{i} \times R_{i}}$$

11 $$\Delta R_{i}=R_{i}\,-\,R_{i-1},\,R_{0}=0,\,P_{0}=2$$

12 $$R=\frac{{{T_{\text{P}}}}}{{{T_{\text{P}}}+{F_{\text{N}}}}}$$

13 $$P=\frac{{{T_{\text{P}}}}}{{{T_{\text{P}}}+{F_{\text{P}}}}}$$

In the formula, R represents the Recall, and P represents the Precision. T_p_, F_p_, and F_n_ denote the number of true positives (instances where the model correctly identifies positive examples), false positives (instances where the model incorrectly identifies negative examples as positive), and false negatives (instances where the model incorrectly identifies positive examples as negative), respectively.

To assess the model’s classification capabilities, this paper selects three metrics: Top-1 Accuracy, Top-5 Accuracy, and the amount of floating-point operations. The specific calculation formulas for Top-1 Accuracy and Top-5 Accuracy are defined as follows:

$$Top\,-\,1\,Accuracy\,=\,\frac{{{T_1}}}{N}$$

$$Top\,-\,1\,Accuracy\,=\,\frac{{{T_5}}}{N}$$

Where T_1_, F_5_ are the number of samples where the correct labeling is not the best probability, and the number of correct labels included in the top five classification probabilities of all test images, respectively.

### Ablation experiment

To validate the effectiveness of the AHC-YOLO algorithm proposed in this paper, the experiments are divided into two parts for verification and analysis, namely detection and classification.

Since the AHC-YOLO algorithm proposed in this paper is an improvement based on the YOLOv8 algorithm, we have chosen to incrementally integrate the four proposed modules into the network for testing.

During the experiments, we ensured that the dataset and hyperparameters remained constant when introducing different improvements. The weight files obtained when the performance reached a stable state were selected as the final training results of the algorithm. The specific experimental data is shown in Table 1.

**Table 1:** AHC-YOLO detection ablation experiment.

Serial	Baseline	EfficientNetV2	DAttention	CSPPC	Parameters/M	mAP	GFLOPs
1	√				3.012	0.867	8.2
2	√	√			2.295	0.882	6.7
3	√	√	√		2.295	0.905	6.7
4	√	√	√	√	1.862	0.911	5.8

As shown in Table 1, by incorporating the four improvements proposed in this paper into the YOLOv8 algorithm, the average precision continuously increased, and the structure also leaned more towards lightweight design. The mAP was improved from 0.867 of the YOLOv8 algorithm to 0.917, an increase of 0.05. Meanwhile, the GFLOPs were reduced from 8.2 to 5.8.

During the ablation study of the AHC-YOLO algorithm, HGNetv2 network was first added to the backbone network, which significantly reduced the algorithm’s parameter volume and also improved the accuracy of the algorithm to some extent. Then, the MLCA attention mechanism was introduced to enhance the algorithm’s focus on the insulator sheds. The computational cost was low, and the parameter volume increased minimally. However, it did improve the accuracy to a certain extent, with the mAP being improved from 0.882 to 0.905. Subsequently, the CSPPC lightweight convolutional structure replaced the C2f module in the Head part, further reducing the algorithm’s parameter volume and GFLOPs while maintaining accuracy. Finally, the loss function was changed to Inner-WIoU, which once again improved the detection accuracy of the insulator sheds. The final mAP of the AHC-YOLO algorithm was improved to 0.917, an increase of 5.77% compared to the original YOLOv8’s 0.867. The GFLOPs were reduced from the original YOLOv8’s 8.2 to 5.8.

In image classification tasks, the Head of the YOLOv8 algorithm is primarily responsible for predicting class probabilities without the need to predict bounding boxes. Therefore, the design of the Head for classification tasks differs in structure and function from that of detection tasks. The Head for classification tasks is more simplified, focusing on class output and not involving the C2f module. To verify the effectiveness of the AHC-YOLO algorithm in classification tasks, we integrated the HGNetv2 network and MLCA attention mechanism on the basis of YOLOv8 and trained the model while keeping other parameters unchanged. The experimental results are shown in Table 2.

**Table 2:** AHC-YOLO classification ablation experiment.

Serial	Baseline	EfficientNetV2	DAttention	Parameters /M	Top-1 Accuracy	Top-5 Accuracy	GFLOPs
1	√			1.443	0.881	1	3.3
2	√	√		0.874	0.897	1	1.9
4	√	√	√	0.874	0.925	1	1.9

### Comparison experiment

To demonstrate the advantages of the AHC-YOLO algorithm in detection and classification tasks, it was compared with other similar algorithms. Initially, a comparative experiment was conducted to detect insulator sheds, involving algorithms such as the YOLO series, RetinaNet network^28^, Faster RCNN^29^, and RT-DETR algorithm^16^. These algorithms were trained on the insulator hydrophobicity dataset proposed in this paper. The results are shown in Table 3.

**Table 3:** AHC-YOLO detection comparison experiment.

Arithmetic	Parameters/M	mAP	GFLOPs
YOLOv5	2.510	0.796	7.1
YOLOv6	4.239	0.847	11.9
YOLOv8	3.012	0.867	8.1
RT-DETR	29.278	0.862	105.2
RetinaNet	40.8	0.775	48.8
Faster RCNN	41.3	0.803	251.4
AHC-YOLO	1.862	0.917	5.8

According to the data in Table 3, it is evident that during detection tasks, although the mAP values of all compared algorithms exceeded 0.77, the AHC-YOLO algorithm still holds a leading position. In terms of lightweight design, the AHC-YOLO algorithm also stands out, with significantly lower GFLOPs and parameter count compared to other algorithms. This demonstrates the algorithm’s advantage in the detection task of composite insulator sheds and meets the requirements for the recognition of composite insulator sheds.

In the task of hydrophobicity level classification, this paper compares the YOLO series algorithms, RepViT algorithm^30^, ResNet algorithm^31^, and the Visual Geometry Group 16-layer network (VGG16) algorithm^32^. The results of these algorithms after training and testing are shown in Table 4.

**Table 4:** AHC-YOLO classification comparison experiment.

Arithmetic	Parameters/M	Top-1 Accuracy	GFLOPs
YOLOv5	1.221	0.858	3.0
YOLOv6	2.966	0.821	8.0
YOLOv8	1.443	0.881	3.3
ResNet	1.335	0.862	3.2
VGG16	138.36	0.803	15.47
RepViT	2.814	0.857	7.0
AHC-YOLO	0.874	0.925	1.9

The data presented in Table 4 indicates that during classification tasks, the AHC-YOLO algorithm has lower GFLOPs and parameter counts compared to other similar algorithms, while leading in Top-1 accuracy. This fully confirms the algorithm’s advantage in the classification task of hydrophobicity levels for composite insulators.

To validate the recognition capability of the insulator sheds in detection tasks by the AHC-YOLO algorithm proposed in this study, we employed heatmaps to illustrate the areas where the model focuses its attention and analyzed the depth of colors in the heatmaps through the visualization of feature maps. The depth of the color directly indicates the magnitude of the regional weight; the deeper the color, the greater the impact of that area on the network’s response, and the more significant its contribution. Heatmap visualization was implemented by computing layer-wise activation magnitudes across the test dataset, followed by spatial aggregation and normalization. The visualization pipeline was developed using Python 3.9.0.

**Fig. 7:**
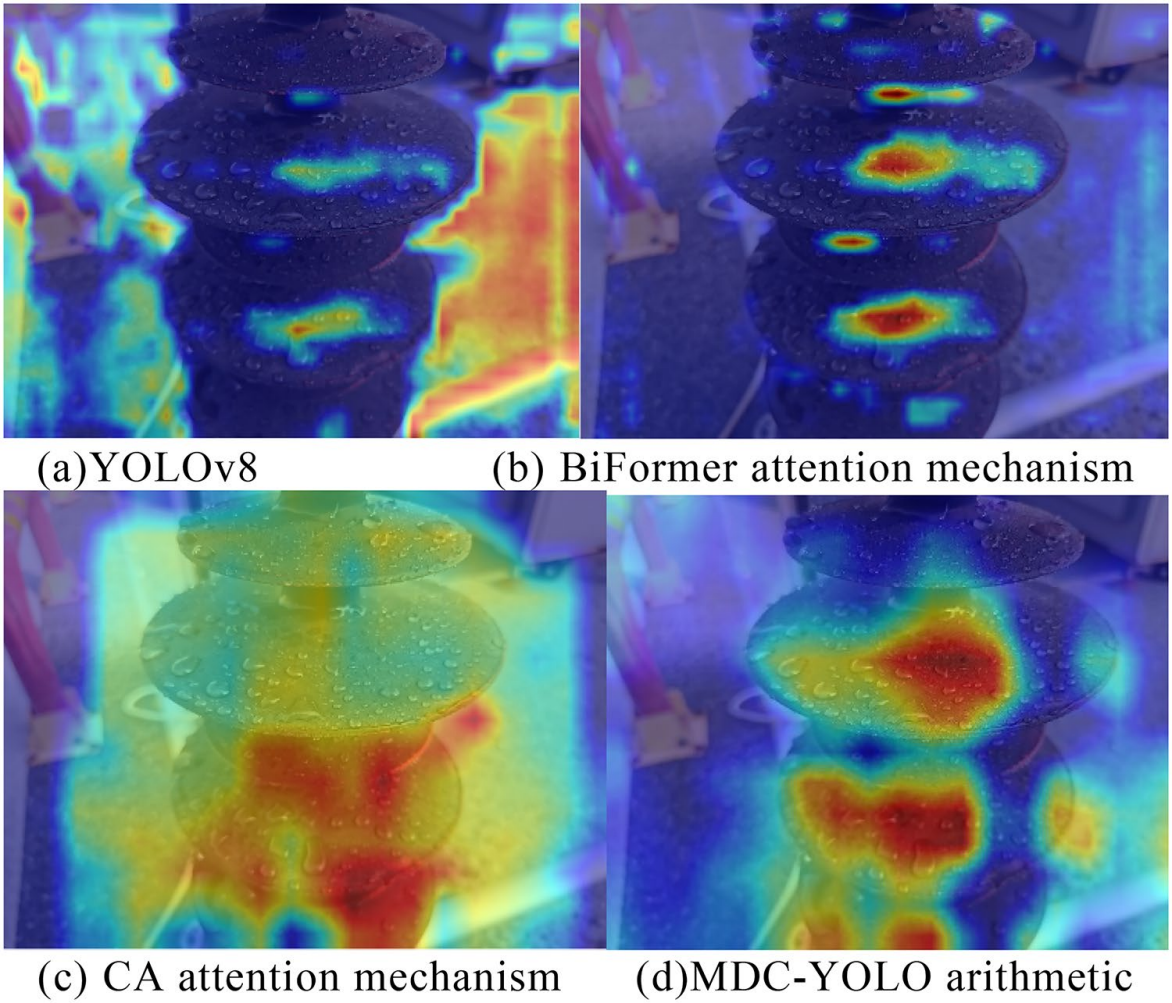
The heat map of different network outputs. The heatmap was generated using a custom Python script (version 3.9.0, https://www.python.org/downloads) developed by our team.

By analyzing the heatmaps in Fig. 7, the performance of different networks can be assessed. Initially, Fig. 7(a) presents the heatmap of the YOLOv8 algorithm without the introduction of an attention mechanism. The heatmap indicates unusually high attention in certain areas, but these areas do not focus on the target. Subsequently, Fig. 7(b) displays the heatmap of the YOLOv8 algorithm after incorporating the Bi-Level Routing Attention (BiFormer)^33^ mechanism, where the algorithm’s focus has shifted to the composite insulators, yet the attention is confined to a limited area. Figure 7(c) shows the heatmap of the YOLOv8 algorithm integrated with the Coordinate Attention (CA)^34^ mechanism, and although there is some concentration of attention on the composite insulators, the overall attention is rather dispersed, with an overly broad scope. Ultimately, Fig. 7(d) exhibits the heatmap of the algorithm proposed in this study, where the majority of the attention is concentrated on the composite insulators, demonstrating a significant weight proportion allocated to them, thereby validating the effectiveness of the improvements made in the attention mechanisms of this algorithm.

## Conclusion

In this study, a YOLO algorithm targeted at the precise detection of the hydrophobicity of composite insulators has been proposed. This algorithm effectively addresses the challenge of extracting hydrophobicity features from complex on-site insulator images. Ablation and comparative experiments have led to the following conclusions:

1) This research utilized drones to spray and photograph de-energized composite insulators, thereby expanding the dataset to better reflect real-world conditions and achieving dataset balance.

2) The proposed AHC-YOLO algorithm, based on the original YOLOv8 algorithm, has achieved dual optimization in accuracy and lightweight design. In terms of recognizing the sheds of composite insulators, the algorithm increased the mean Average Precision (mAP) by 5.19%, while reducing GFLOPs to 3.5. For the task of classifying the hydrophobicity levels of the sheds, Top-1 accuracy was improved by 4.654%, with GFLOPs reduced to 1.1.

3) Experimental validation has shown that the AHC-YOLO algorithm outperforms seven other algorithms in both detection and classification tasks, in terms of both accuracy and lightweight design. The algorithm is capable of effectively identifying the sheds of composite insulators and classifying their hydrophobicity levels. Due to its lightweight characteristics, it is suitable for deployment on edge devices such as drones, expanding its range of applications.

## Data Availability

The data that support the findings of this study are available from the corresponding author upon reasonable request.
